# Electrical conductivity of the global ocean

**DOI:** 10.1186/s40623-017-0739-7

**Published:** 2017-11-14

**Authors:** Robert H. Tyler, Tim P. Boyer, Takuto Minami, Melissa M. Zweng, James R. Reagan

**Affiliations:** 1 Geodesy and Geophysics Laboratory, Code 61A, NASA Goddard Space Flight Center, Greenbelt, MD 20771 USA; 2 Astronomy Department, University of Maryland, College Park, MD USA; 3 National Centers for Environmental Information (NCEI), National Oceanic and Atmospheric Administration (NOAA), Washington, DC USA; 4 Earthquake Research Institute, The University of Tokyo, 1-1-1 Yayoi, Bunkyo-ku, Tokyo, 113-0032 Japan; 5 Cooperative Institute for Climate and Satellites (CICS), Earth System Science Interdisciplinary Center (ESSIC) University of Maryland, College Park, MD USA

**Keywords:** Electrical conductivity, Conductance, Ocean, Climatology

## Abstract

The electrical conductivity of the ocean is a fundamental parameter in the electrodynamics of the Earth System. This parameter is involved in a number of applications ranging from the calibration of in situ ocean flow meters, through extensions of traditional induction studies, and into quite new opportunities involving the remote sensing of ocean flow and properties from space-borne magnetometers such as carried aboard the three satellites of the *Swarm* mission launched in 2013. Here, the first ocean conductivity data set calculated directly from observed temperature and salinity measurements is provided. These data describe the globally gridded, three-dimensional mean conductivity as well as seasonal variations, and the statistics of spatial and seasonal variations are shown. This “climatology” data set of ocean conductivity is offered as a standard reference similar to the ocean temperature and salinity climatologies that have long been available.

**Electronic supplementary material:**

The online version of this article (10.1186/s40623-017-0739-7) contains supplementary material, which is available to authorized users.

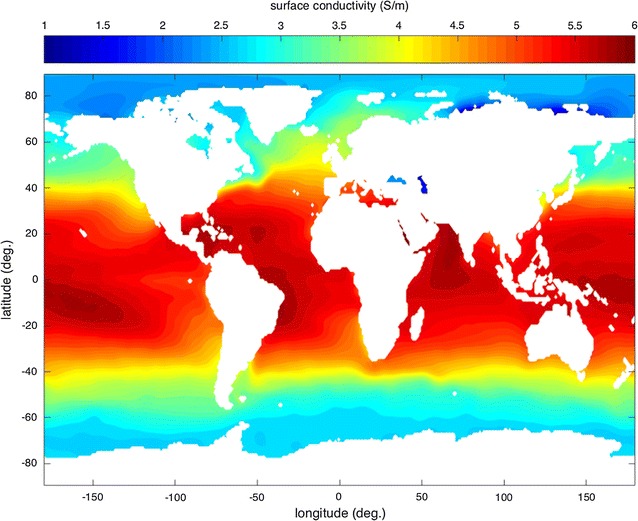

## Introduction

The electrical conductivity of the ocean is a fundamental parameter in the electrodynamics of the Earth System. It affects electrodynamic processes and the observable electromagnetic fields within the ocean but also throughout the Earth’s various components extending from the interior to the upper atmosphere. Knowledge of the ocean’s electrical conductivity is therefore required in studies of these processes and interpretation of these fields.

The electrical conductivity referred to in this article is specifically the frequency-independent proportionality constant $\sigma$ relating electrolytic conduction current density ${\mathbf {J}}$ to the electric field ${\mathbf {E}}'$ as measured in a frame moving with the fluid (the units of conductivity are Siemens/meter (i.e., S/m)). This relationship is expressed as Ohm’s law ${\mathbf {J}}=\sigma {\mathbf {E}}'$. Using the Lorentz transformation, this can also be written as ${\mathbf {J}}=\sigma \left( {\mathbf {E}}+{\mathbf {u}}\times {\mathbf {B}}\right)$, where ${\mathbf {E}}, {\mathbf {u}}$, and ${\mathbf {B}}$ are the electric field, fluid velocity, and magnetic field measured in the common rotating frame of the Earth. Maxwell’s equations provide further relationships to complete a closed set of equations from which general solutions can be found, but Ohm’s law alone establishes the importance of $\sigma$ in opportunities attempting to infer components of $\sigma , {\mathbf {J}}, {\mathbf {E}}, {\mathbf {u}}, {\mathbf {B}}$ from an incomplete set of observations.

A wide variety of oceanographic instruments infer flow velocity from in situ measurements of the electric field (see Tyler et al. 1997; Szuts 2012 for review material) or, more accurately, the difference in electric potential between two points which, depending on the experimental design, may be separated by distances ranging from centimeters to hundreds of kilometers. The inference of flow from electric field measurements requires estimates of the electrical conductivity distributed over a region typically much larger than can be co-sampled in the same experiment with conductivity sensors. Hence, a database for accurately prescribing the conductivity distribution immediately improves the calibration of a wide variety of oceanographic flow meters.

Since long, processes in the upper atmosphere and magnetosphere have been inferred from observed fluctuations in the geomagnetic field. Electric currents excited by dynamical processes in these regions cast magnetic fields reaching land and satellite magnetic observatories. But these magnetic fluctuations also induce electric currents in the oceans and Earth, and so the observed magnetic fields are due to a combination of the primary and induced electric currents. Accurate inference of the processes in the upper atmosphere and magnetosphere therefore depends on accurate knowledge of the conductivity distribution in the oceans and mantle.

Until relatively recently, most observations of the geomagnetic field were made on land. Since 1999, however, a number of long-term, low-orbit magnetic survey missions (Oersted, CHAMP, SAC-C) have provided unprecedented resolution, particularly over the oceans and other regions poorly covered by land observatories (e.g., Olsen et al. 2006). In 2013, three low-orbit satellites were launched in the first mission (*Swarm*, e.g., Friis-Christensen et al. 2008) involving multiple space-borne magnetic observatories aimed to further increase the detail in the observed magnetic fields. This new epoch of satellite magnetic surveys has opened new opportunities, some extending from the applications described above, and some quite novel. Many of these opportunities involve interpretation of large-scale, relatively weak fluctuations in the Earth’s magnetic field that in turn involve electric currents (either primary or induced) in the ocean (e.g., see Kuvshinov 2008 for review). Hence, much of the modern development in geomagnetic studies has become dependent on the accuracy in modeling oceanic electric currents, and the priority for better understanding the distribution of electrical conductivity in the ocean has risen.

In the following two subsections, the physics of ocean electrical conductivity is described, and a description of the methods used in previous estimates of this parameter is given. The methodology of this study is presented in “Methodology” section, and the results are presented in “Climatology of ocean electrical conductivity” section. In “Use of climatology to assess errors in previous assumptions” section, a brief description of errors associated with previous conductivity estimates is provided, a discussion is included in “Conclusion” section, and information on obtaining the data is given in “Availability of data and materials” section. This data set of the long-term (1981–2010) climatological mean shall be referred to here as the “climatology,” consistent with nomenclature for other ocean data.

### Electrical conductivity of seawater

Within the parameters of Earth’s oceans, the electrical conductivity of seawater depends on temperature, salinity, and to a much smaller degree pressure (depth). Salts such as sodium chloride (NaCl) disassociate in water to form cations ($\hbox {Na}^{+}$) and anions ($\hbox {Cl}^{-}$) that migrate in the presence of an electric field, thereby producing an electric current. It is then easy to understand that the conductivity $\sigma$ increases with the concentration of dissolved salts (salinity *S*). The conductivity also increases with increasing temperature *T*, and this may be reasonably associated with an increase in mobility of the ions. Although the increase of $\sigma$ with *T* and *S* may be easily understood intuitively, this does not immediately provide much indication of the distribution of $\sigma$ in the ocean, even for oceanographers familiar with the distributions of *T* and *S*. Even the typical vertical profile of $\sigma$ is not easily anticipated because of opposing tendencies in the dependencies of conductivity and density on *T*, *S*. While $\sigma$ *increases* with both *T* and *S*, the water density $\rho$ *increases* with *S* but *decreases* with *T*. With possible exceptions that are volumetrically rare (e.g., surf zones), the oceans are stably stratified and $\rho$ increases with depth. This alone, however, does not immediately provide intuition on the profile of $\sigma$ or whether the profile is even monotonic with depth. It seems then to be the case that quantitative and even qualitative understanding of the distribution of ocean conductivity must be obtained through original analyses of observations, rather than through reference to other well-described ocean parameters.

### Global conductivity in previous studies

In early studies, and even continuing to recent work (e.g., Tyler et al. 2003; Kuvshinov et al. 2006; Schnepf et al. 2014; Sabaka et al. 2015), the electrical conductivity of the ocean has often been assumed to be uniform. In some applications involving idealized or model comparison studies, this may be justified but it is also clear that a reliable global description or gridded data set of the observed ocean conductivity was not available. Tyler et al. (1997) and several subsequent studies extending to the present (e.g., Irrgang et al. 2016a, b) included a description of the three-dimensional ocean conductivity as obtained from the temperature, salinity, and pressure variables in a global ocean circulation model. While the conductivity obtained this way is expected to be dynamically consistent, it remains unclear how realistic the description is and, most importantly, how well it agrees with the large set of scattered observations.

Climatology data sets for other ocean parameters (e.g., temperature, salinity, density) have long been available and used in a broad variety of oceanographic applications ranging from observational studies to ocean modeling. But a climatology data set for conductivity has not previously been available and construction of such requires a major effort. Climatologies of ocean temperature and salinity, for example, attempt to represent the most reliable gridded data sets that can be constructed from the large set of historical observations taken using a variety of methods and instrumentation. Conductivity depends nonlinearly on temperature and salinity, and the separate temperature/salinity climatologies are constructed from quite different sample distributions. Conductivity should then be calculated from co-observed temperature and salinity.

Alternatively, one may calculate a description of conductivity directly from climatologies of temperature and salinity. Such an approach was used to obtain the conductivity in Manoj et al. (2006), and these data have been adopted in later studies (e.g., Kuvshinov 2008; Sabaka et al. 2015; Schnepf and Kuvshinov 2015; Grayver et al. 2016). While this may reasonably provide a quick approach to obtaining a gridded conductivity data set, a major weakness is that the realism is immediately suspect and the uncertainties are not easily assessed. The only published study so far to include gridded conductivity calculated from observations is the Tyler tidal magnetic field simulation in Sabaka et al. (2016). The latter data (a preliminary data set of that presented in this study) used data from the longer range 1978–2012, and this date range was adjusted to 1981–2010 in this study to follow the time span convention of other climatologies.

As described, previous descriptions of global ocean conductivity are either recognizably simplistic, or their degree of realism is difficult to assess. Of course with the climatology provided here, the errors associated with previous approaches can now be addressed, and a discussion toward this is included in “Use of climatology to assess errors in previous assumptions” section.

## Methodology

In this section, the algorithm used for calculating seawater electrical conductivity from temperature, salinity, and pressure is first presented, and then the data serving as input is described.

### Algorithm for calculating electrical conductivity

The electrical conductivity ($\sigma$) of seawater became one of the fundamental oceanographic parameters in the 1950s with the increased use of high-precision electrical conductivity bridges for salinity determinations. After almost 70 years of using the dilution theory provided by Knudsen et al. (1902), the practical salinity (*S*) was defined on the Practical Salinity Scale of 1978 (PSS-78) in terms of the conductivity ratio to the reference, and its relationship with $\sigma$, temperature ($t_{68}$, IPTS-68), and pressure (*p*, zero dbar at one atmospheric pressure) was incorporated in the Equation of State for Seawater (EOS-80; e.g., Fofonoff and Millard 1983; Fofonoff 1985). Although the international thermodynamic equation of seawater 2010 (TEOS-10; IOC et al. 2010) superseded EOS-80, the relationship in EOS-80 associated with the conductivity is still valid. In this study, we use **gsw_C_from_SP** in the Gibbs Seawater (GSW) Oceanographic Toolbox (McDougal and Barker 2011; IOC et al. 2010) to calculate $\sigma$ from *S*, *p*, and the temperature on ITS-90, *T* (TEOS-10 is used with no conversions between temperature scales; if data were in IPTS-68 or ITS-90, we used it directly). In both EOS-80 and TEOS-10, the conductivity, $\sigma$, is calculated using the relationship,

1 $$\begin{aligned} \sigma (S,t_{68},p)=\sigma (35,15\,^{\circ }\mathrm{C},0\;\mathrm{dbar})\cdot R, \end{aligned}$$

where *R* is the solution of

$$\begin{aligned} r_{t}(t_{68})\cdot R_{t}\cdot \left( 1+\frac{C_{p}(p)}{A(t_{68})\cdot R+B(t_{68})}\right) -\,R=0, \end{aligned}$$

and $R_{t}$ is obtained by solving the following equation:

2 $$\begin{aligned} \left\{ \sum _{i=0}^{5}\left( a_{i}+\frac{t_{68}-15}{1+k(t_{68}-15)}b_{i}\right) (R_{t})^{i/2}\right\} -\,S=0. \end{aligned}$$

Here, $r_{t}(t_{68})=\sum _{i=0}^{4}c_{i}t_{68}^{i}, B(t_{68})=1+d_{1}t_{68}+d_{2}t_{68}^{2}, A(t_{68})=d_{3}+d_{4}t_{68}$, and $C_{p}(p)=e_{1}p+e_{2}p^{2}+e_{3}p^{3}$, while $k, a_{i}, b_{i}, c_{i}, d_{1\ldots 4},$ and $e_{1\ldots 3}$ are empirical coefficients.

The reference conductivity is $\sigma (35,15\,^{\circ }\mathrm{C},0\;\mathrm{dbar})=4.29140$ (S/m). Between the two temperature scales, IPTS-68 and ITS-90, *T* is linked to $t_{68}$ by the relationship, $t_{68}= 1.00024 \cdot T$. While Eq. (1) is valid only in the range of $2<S<42$, GSW Toolbox adopts the method of Hill et al. (1986) for the range of $0<S<2$. Refer to Fofonoff (1985) and IOC et al. (2010) for more details about Eq. (1).

Concurrent salinity, temperature, and pressure measurements used to calculate conductivity come from the World Ocean Database (hereafter WOD). Descriptions of data sources for this collection of oceanographic profile data, instrumentation, temporal and spatial distributions, measurement accuracies, and quality control procedures can be found in Boyer et al. (2013). Only concurrent salinity and temperature profiles taken during or after 1981 were used for consistency in the definition of salinity (PSS-78). Pressure was used when reported; otherwise, it was calculated from reported depth measurements. Although most salinity values in this time period are derived from conductivity measurements, the conductivity was not usually reported, hence the need to back calculate conductivity. The same procedures as outlined in Zweng et al. (2013) were followed to calculate objectively analyzed climatological mean fields of conductivity for the period 1981–2010 at 102 standard depth levels from the surface to 5500 m depth. Briefly, one-degree gridded mean values of conductivity at each standard depth were compiled after back calculation, subject to quality control procedures. An objective analysis technique (Cressman 1959; Barnes 1964) was employed to modify each one-degree mean based on the difference between a first-guess field and the compiled mean of each one-degree square within a given radius of influence. The first-guess field for the annual (all-data) field was a basin specific zonal average. The first-guess field for the four seasonal fields (Winter = January–March, etc.) was the annual climatological mean. The first-guess for each month was the appropriate seasonal climatological mean. Monthly fields only extend to 1500 m, as there is little annual cycle (and sparse data) below that level. Annual and seasonal fields extend to 5500 m depth.

## Climatology of ocean electrical conductivity

In this study, the temporal mean ocean conductivity data for the global ocean have been calculated following the method described in “Methodology” section. We proceed here to describe characteristics of this data set which is available at https://www.nodc.noaa.gov/OC5/woa13/.

The temporal mean conductivity $\sigma =\sigma (\text{ longitude, } \text{ latitude, } {-\,z) }$ is a three-dimensional array, with each value representing a cell volume which varies with latitude and depth ($-\,z$). The volumetrically weighted mean conductivity of the global ocean is 3.31 ± 0.23 S/m.

**Fig. 1:**
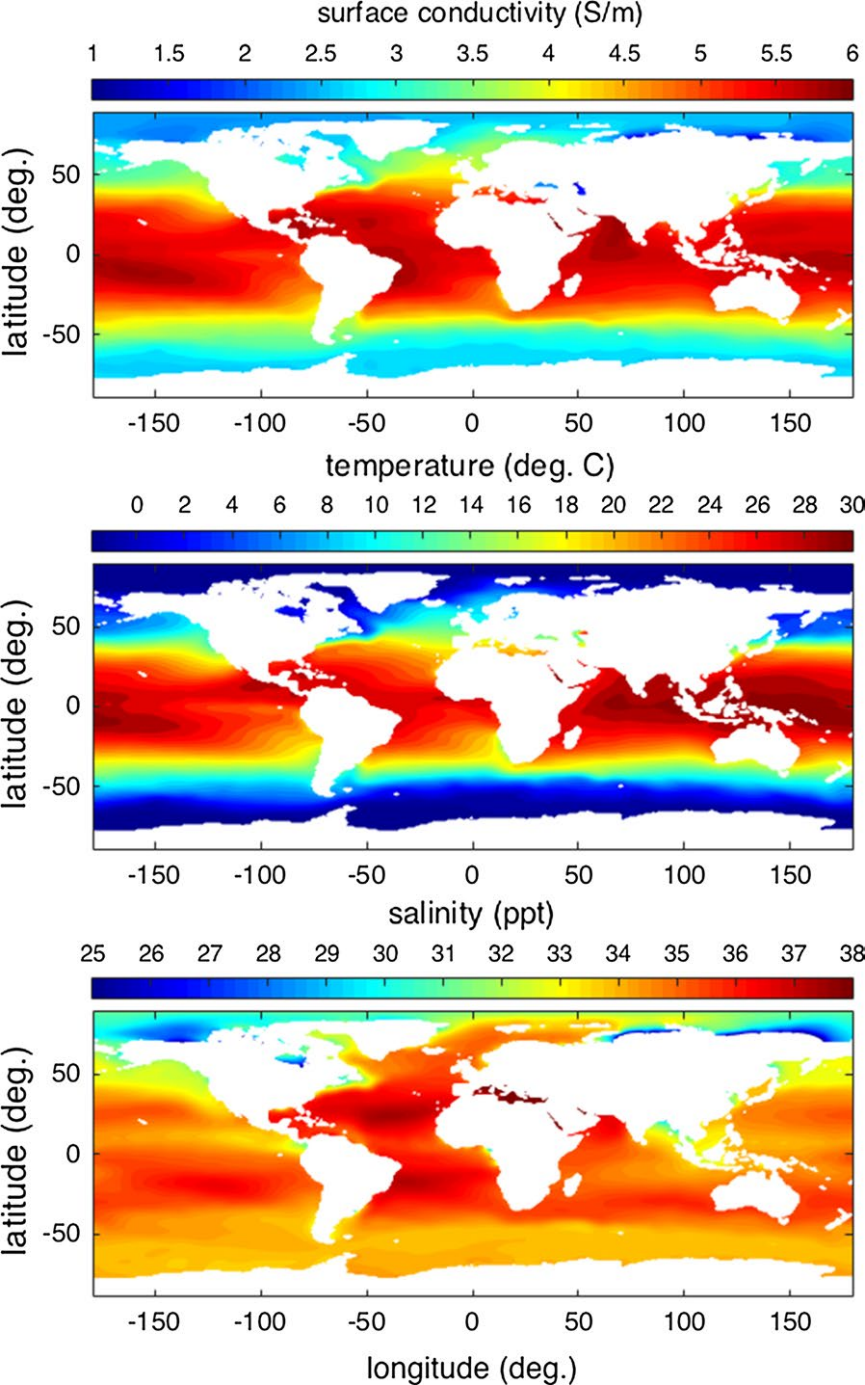
Electrical conductivity, temperature, salinity, and density at the surface of the global ocean. The full ranges in the surface data are, respectively, 0.0999–6.45 (S/m), − 1.84–30.6 (C), 5.02–40.1 (ppt)

In Fig. 1, we see that the values and variability of the conductivity at the ocean surface are much larger than the volume quantities. There is a strong latitudinal variation that primarily tracks that of temperature. But in fresh inland seas and locations near the mouths of rivers, conductivity primarily depends on salinity. The salinity dependence is quite extreme in the Arctic because the temperature is relatively constant (near freezing), there are many large rivers supplying freshwater, and brine rejection/salt dilution during ice formation/melt makes the variability of salinity high. Because evaporation exceeds precipitation over the Mediterranean (and generally the Atlantic), both salinity and conductivity are elevated compared to other locations at similar temperatures.

**Fig. 2:**
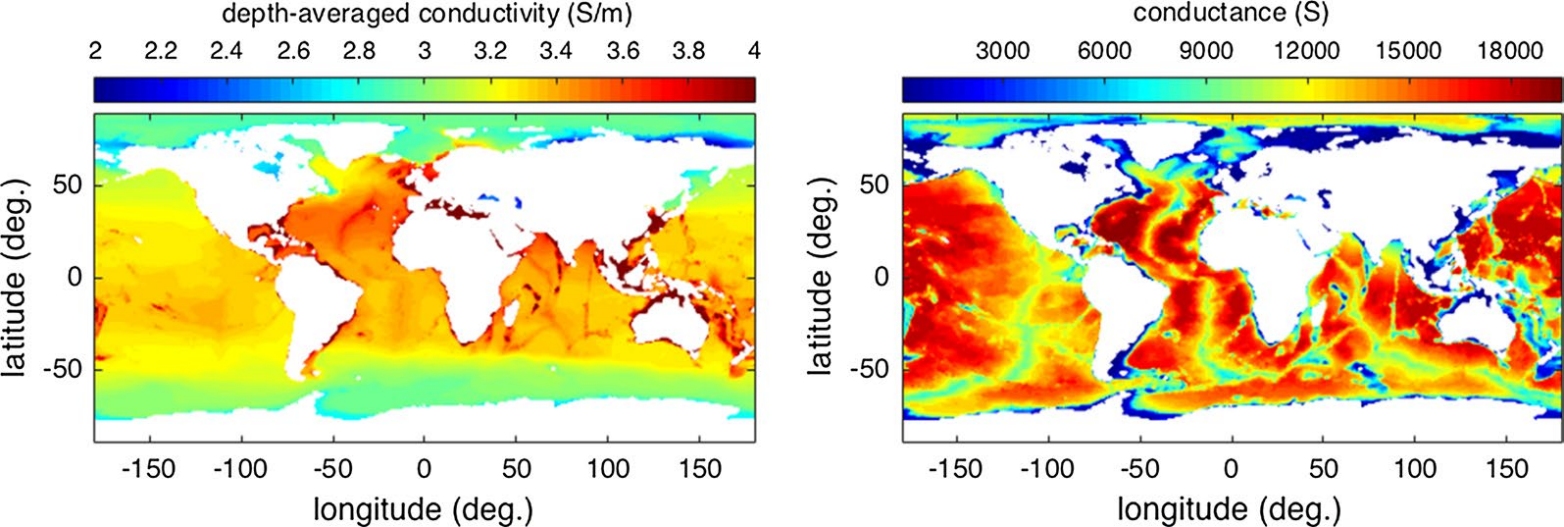
Depth-averaged electrical conductivity and conductance of the global ocean. The full range in the data is, respectively, 0.212–6.36 (S/m) and 0–1962 (S)

**Fig. 3:**
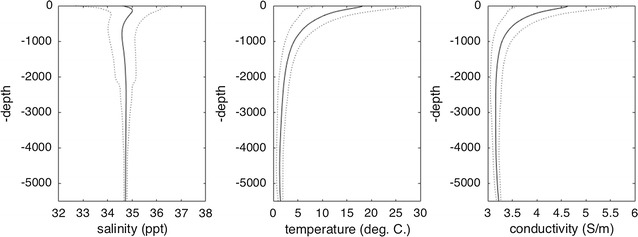
Globally averaged conductivity profile with depth (profiles for temperature and salinity shown for comparison). Dash lines show the envelope of one standard deviation

The depth-averaged conductivity is shown in Fig. 2. Away from warm, shallow areas and the freshwater of rivers and inland seas, one sees that the depth-averaged conductivity is remarkably constant over most of the global ocean. This is an immediate indication that most of the variability seen in Fig. 1 does not extend very deep into the ocean. The related depth integral (i.e., the depth-averaged conductivity multiplied by the ocean depth) is referred to as the conductance (S) and is also shown in Fig. 2. The globally averaged profile of conductivity with depth is shown in Fig. 3, together with similar profiles for *T*, *S*.

**Fig. 4:**
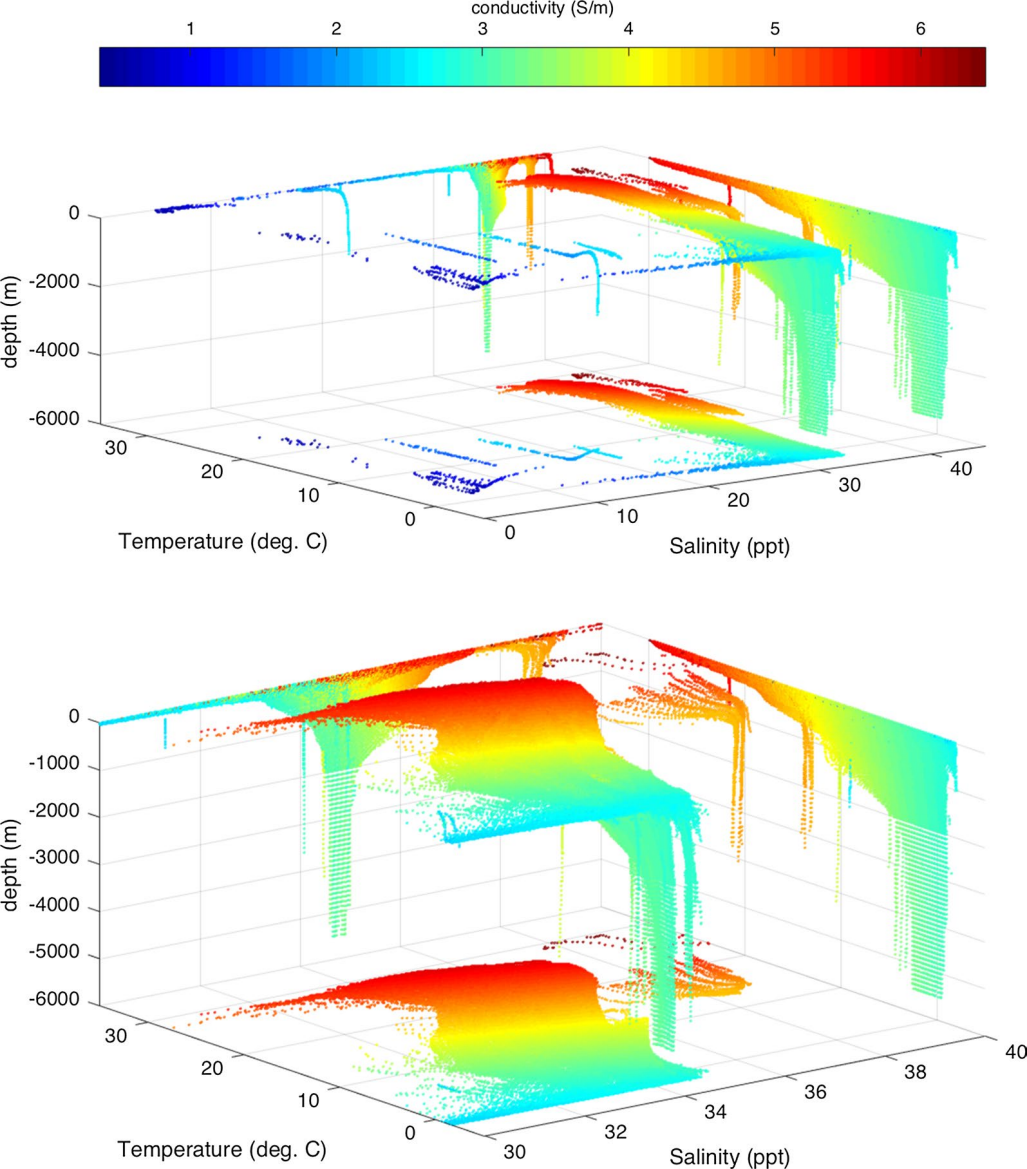
Scatter plot showing data set of global ocean conductivity (values given by color scale) as distributed in coordinates of co-located salinity, temperature, and depth. Planar projections are also included. Clusters describe distinct water mass properties from various ocean bodies. The bottom panel is a magnification of the top panel, excluding the atypical low-salinity clusters associated with inland seas (see text). Most of the variability is near the ocean surface

To characterize the three-dimensional behavior of the conductivity, we follow an approach used in oceanography in which *T*, *S* are used as plotting coordinates. Because a water mass’ *T*, *S* properties are primarily set by processes occurring at the sea surface, the *T*, *S* coordinates are often useful in identifying the location of origin of the water mass. In Fig. 4, a scatter plot of the conductivity data is shown using *T*, *S* (and depth, $-\,z$) as coordinates. The value of the conductivity $\sigma \left( T,S,-\,z\right)$ is shown by the color scale. Several projections onto planar surfaces are included and used toward the following description.

Consider the projection onto the $-\,z=6000$ m surface (as seen in the top panel of Fig. 4). Several point clusters with low *S* coordinate correspond to inland seas (Caspian, Black, Baltic) and the Arctic. Excluding these regions, we magnify this figure (bottom panel) and see that aside from a cluster with high *T*, *S* (the Mediterranean), most of the global ocean conductivity is remarkably uniform at depth (see also the Additional file 3 which shows a movie of conductivity surfaces from the surface to seafloor).

**Fig. 5:**
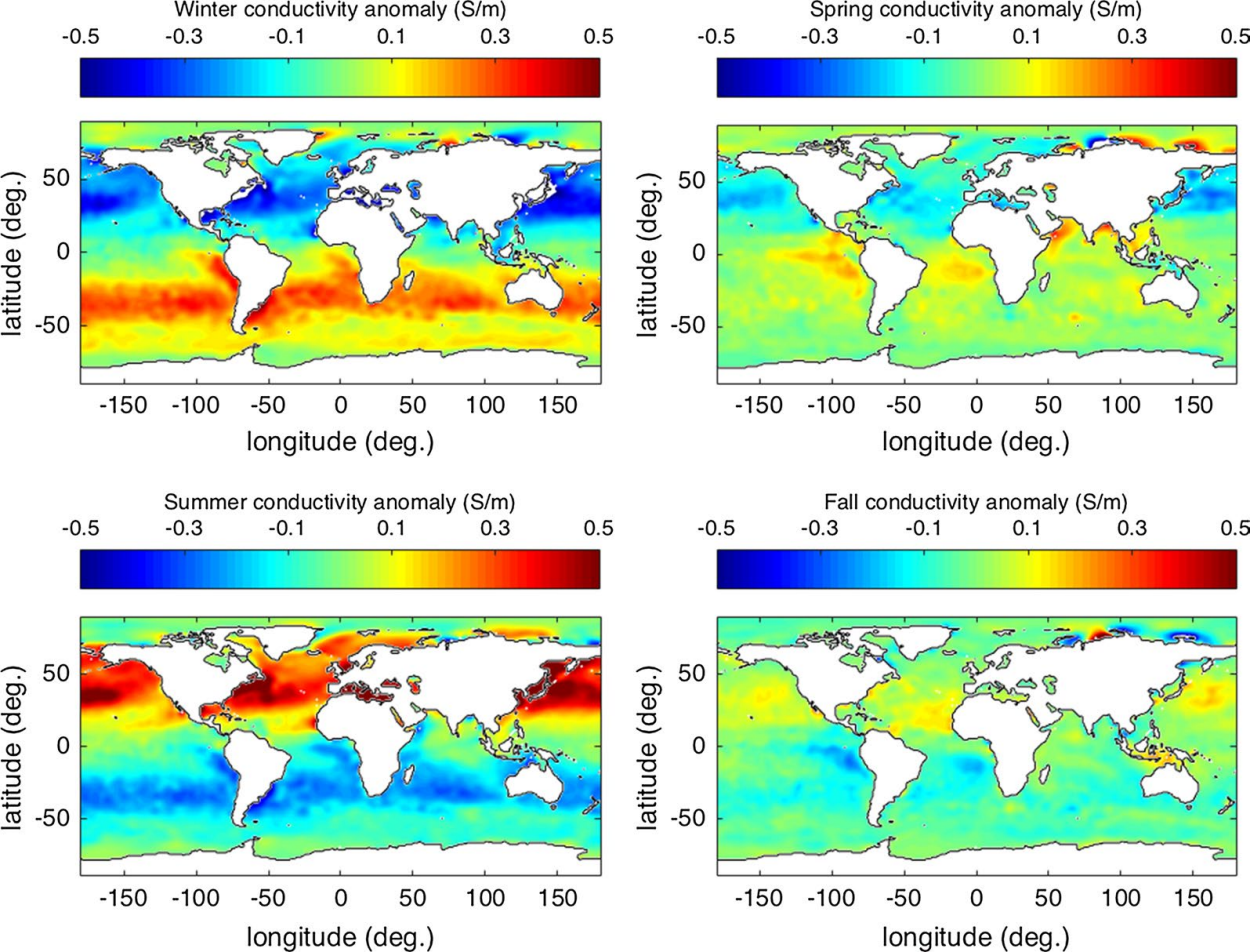
Ocean surface conductivity anomaly (relative to annual mean) for each of the four seasons. The full range in the data is for Winter, Spring, Summer, and Fall, respectively, − 0.852–0.520 (S/m), − 0.626–0.506 (S/m), − 0.633–0.963 (S/m), and − 0.591–0.642 (S/m)

Above, the spatial variability of the temporally averaged conductivity has been described. Now we shall describe the temporal variability. One should note that even the time-averaged conductivity climatology can be useful in understanding and predicting temporal variability caused by fluid motion. Figures 3 and 4 show that there are typically large vertical gradients near the surface. Therefore, vertical fluid motion (e.g., internal waves, convection) can cause temporal variability in the conductivity measured at a given location. Although the horizontal gradients in conductivity are much smaller than the vertical gradients, persistent advection by ocean currents associated with eddies, waves, and tides can also lead to important temporal fluctuations in conductivity. The expected amplitudes of these fluctuations depend very much on the features considered, but a simple estimate is $\Delta \sigma \sim |{\mathbf {u}}\cdot \nabla \sigma |\Delta t$ , where $\Delta \sigma$ is the amplitude of the anomaly, $|{\mathbf {u}}\cdot \nabla \sigma |$ is the amplitude of the dot product of flow velocity ${\mathbf {u}}$ (which may consider vertical or horizontal components) and the gradient of the conductivity, and $\Delta t$ is the time scale for the process. On the large scales relevant to the applications described in this article, the most predominant temporal fluctuation to expect is due to the seasonal changes in the fluxes of heat and freshwater across the ocean boundaries. For the purposes of the large-scale climatology described here, we shall use the seasonal changes to characterize the temporal variability in the ocean’s conductivity. In Fig. 5, the surface values are seen to vary $\sim \,1$ S/m over the seasonal cycle, the fluctuations traceable to expected seasonal variations in heat and freshwater fluxes. The large-scale seasonal variations at the surface show, at mid and low latitudes, a dominant dependence on hemispheric warming/cooling rather than salinity. Regionally (notably the Arctic) conductivity fluctuations can show strong dependence on river runoff.

As shown in Additional file 1: Fig. S1, we see that the depth-averaged temporal fluctuations are typically extremely small $\sim \,0.01$ S/m. The corresponding fluctuation in terms of conductance is $\sim 50$ S. This is consistent with the expectation that most of the seasonal variations in the surface fluxes of heat and freshwater do not penetrate below the top few hundred meters of the ocean.

There are two sources of uncertainty to consider in the conductivity climatologies. The climatological mean value is a single value representing the mean over the given time period at each one-degree grid box. However, there is variation around that mean, both natural variation and that caused by measurement uncertainty. The measurement accuracy for a standard Seabird 911+ CTD instrument is 0.00003 S/m for conductivity and 0.001C for temperature. In practice, due to ocean conditions, CTD calibration, and measurement procedures, the uncertainty in conductivity may be higher. It is difficult to separate natural variations from measurement uncertainty. So the standard error of the mean represents the combined uncertainty of the mean value for each depth at each one-degree grid box. The global mean of the standard error is 0.02 S/m at the surface and 0.003 S/m at 1000 m depth for monthly climatological mean fields and $0.04 \pm 0.03\,\hbox {S/m}, 0.004 \pm 0.004$ S/m at the surface and 1000 m, respectively, for the annual climatological mean field. The annual mean field has a higher standard error, as expected, as it encompasses the entire seasonal cycle. The second measure of uncertainty is the difference between the observed mean and the objectively analyzed mean at each one-degree grid box. This is a measure of the uncertainty introduced in the objective analysis procedure. It is not an independent measure from the standard error of the mean, as the natural variations and discrepancies in measurement accuracy play a role in the differences between values in nearby grid boxes and hence are partially responsible for the difference between observed and analyzed mean fields. For monthly fields at the surface, the global mean observed minus analyzed is approximately $0.003 \pm 0.08$ S/m, while at 1000 m the value is $0.0004 \pm 0.02$ S/m. For the annual time period at the surface, the global mean observed minus analyzed difference is $0.006 \pm 0.09$ S/m at the surface and $0.0005 \pm 0.01$ S/m. All values are for the 1981–2010 climatological mean fields of conductivity. Full fields of standard error of the mean and observed minus analyzed difference are provided with the climatological mean fields of conductivity at https://www.nodc.noaa.gov/OC5/woa13/.

## Use of climatology to assess errors in previous assumptions

As described in “Introduction” section, a weakness in previously assumed ocean conductivity distributions was the difficulty in assessing the realism or associated errors in the estimates. With the climatology provided here, one may now assess these errors. Although some comments and comparisons are included here, it should be noted that with the climatology data now available there is little reason to justify the continued use of simplistic assumptions. The discussion here then serves only to address errors due to assumptions used in previous applications. As this is not the goal of this paper, the discussion here is kept brief. Because accuracy in the previous applications can depend on not just the values of the conductivity but also the gradients and integrals, it is also clear that such an error assessment would remain incomplete unless performed by the original investigators using the new conductivity data provided.

As described in “Methodology” section, we calculated the gridded conductivity data set by first calculating conductivities from concurrently measured in situ *T*, *S*, *p* and then conducting the objective analysis against the irregularly distributed conductivity data. One can much more easily obtain a gridded conductivity data set by directly calculating conductivity from existing *T*, *S*, *p* climatologies, as conducted in Manoj et al. (2006). However, the latter conductivity data need not be representative nor even realistic.

The climatological *T*, *S* data in the World Ocean Atlas 2013 (Boyer et al. 2013; Zweng et al. 2013) represent spatial/temporal distributions of observed *T*, *S*, but the distributions of observed *T*, *S* data are somewhat different because some observations reported only one of either *T* or *S*. While climatologies of either *T* or *S* can be obtained through objective analyses of the respective *T*, *S* data available, problems can arrive when one attempts to combine these data derived from different temporal/spatial distributions of original data. This is especially a concern in the case here where the calculation of conductivity involves a nonlinear dependence on *T*, *S*. The latter approach may indeed give conductivity values that are neither representative nor realistic. At the heart of this matter is the fact that the formulae for calculating conductivity from *T*, *S* require that the *T*, *S* measurements are taken at the same time and location. The approach in this study restores this requirement by creating conductivity climatologies from coincident, co-located *T*, *S* observations rather than the processed *T*, *S* climatological products.

Several previous studies treated the ocean as having a uniform conductivity of value 3.2 S/m (Tyler et al. 2003; Kuvshinov et al. 2006; Schnepf et al. 2014; Sabaka et al. 2015). This can be compared with the global mean of 3.31 S/m described in “Climatology of ocean electrical conductivity” section. One also sees in Fig. 2 that assumption of a uniform value is quite crude as there are both regional and zonal departures. Most notably, the Arctic, with its large river runoff, has much lower conductivity due to the reduction in salinity, and a global-scale reduction due to the lower temperatures at high latitudes is also apparent.

While it can be immediately appreciated that the conductivity data presented here are significantly more realistic than the assumption of uniform ocean conductivity, the improvement over the data previously calculated from *T*, *S* climatologies is less immediate to describe and is therefore included in Additional file 2: Supporting Information to this paper. In brief, conductance calculated in the two methods (or even with the uniform conductivity assumption) appears very similar over most of the ocean, but this is primarily simply reflecting the common ocean depth used in all cases. Examination of the gradients of conductance and its inverse show larger fractional differences in the methods. Finally, a better test of the differences (in terms of testing the ocean conductivity distribution assumed rather than common integration limits) is in comparing the depth-averaged conductivity and its gradients (as shown in Additional file 3: Supporting Information). In this latter case, there are large differences between the two methods.

In summary, the results here support idealized ocean conductivity assumptions as an approximation (uniform conductivity multiplied by realistic ocean depth gives a reasonable conductance distribution, and one may even obtain a reasonable depth-averaged conductivity assuming a common conductivity depth profile appropriately representing the global averaged behavior). The conductivity distribution as calculated in Manoj et al. (2006) may similarly serve the same level of approximation but also shows spurious features such that it is recommended that use of these data is discontinued in favor of the data presented here in applications attempting to represent the realistic electrical conductivity of the ocean.

## Conclusion

The generation and analyses of the conductivity climatology data set have shown that the spatial and seasonal variability is remarkably small, at least when compared with the conductivity in other components of the Earth System which may typically show orders of magnitude variations in both space and time. The ocean appears to be the only large-scale component of the Earth System with such highly predictable electrical conductivity, and the data presented here provide a standard reference. This stability and predictability of the ocean conductivity is very important in a number of induction and motional induction applications where naturally occurring magnetic fields (driven by external fields or flow) may be used to infer flow and electrical parameters in the ocean and solid Earth. In some case (e.g., tides) where the forces ultimately driving oceanic electric currents are also highly predictable, the ocean may provide the most predictable naturally occurring large-scale sounding source on Earth. In this case, the data presented here may be used to quantify the stability of such sources.

While the conductivity of the ocean may be more stable and predictable than other components of the Earth System, the climatology presented also shows interesting spatial and temporal variations. Immediately apparent are the effects of seasonal heating and river runoff.

Quantifying errors in past studies due to simplistic assumptions for the conductivity is necessarily incomplete as accuracy can depend on not only the conductivity values used but also the gradients and integrals. Some reassurance in past simplistic assumptions is provided here in that the spatial variability is seen to be relatively small and/or described by roughly similar depth profiles (hence, idealized assumptions are not as bad as they could first seem). But in attempting to represent more realistic ocean conductivity, it is recommended that use of conductivity as calculated in Manoj et al. (2006) be discontinued in favor of the results provided here. Future studies that intentionally continue to use idealized or ocean model conductivity distributions may also now draw from the statistics and data provided here.

## Additional file

**Additional file 1: Figure S1.** Depth-averaged conductivity anomaly (relative to annual mean) for each of the four seasons. The full range in the data are, respectively, −0.660–−0.556 (S/m), −0.471–−0.317 (S/m), −0.511–−0.721 (S/m), −0.502–−0.594 (S/m).

**Additional file 2.** Supplement document comparing results here with results from a previous study.

**Additional file 3.** Movie of ocean conductivity showing frames moving from surface to sea floor.
