# Supplementary material for: Electrical conductivity of the global ocean
Source: Earth Planets Space. 2017 Nov 14;69(1):156. doi: 10.1186/s40623-017-0739-7 (PMC6959386; doi:10.1186/s40623-017-0739-7)
Supplement: Supplementary file 1 — Additional file 1: Figure S1. Depth-averaged conductivity anomaly (relative to annual mean) for each of the four seasons. The full range in the data are, respectively, −0.660–−0.556 (S/m), −0.471–−0.317 (S/m), −0.511–−0.721 (S/m), −0.502–−0.594 (S/m). [file 40623_2017_739_MOESM1_ESM.pdf]

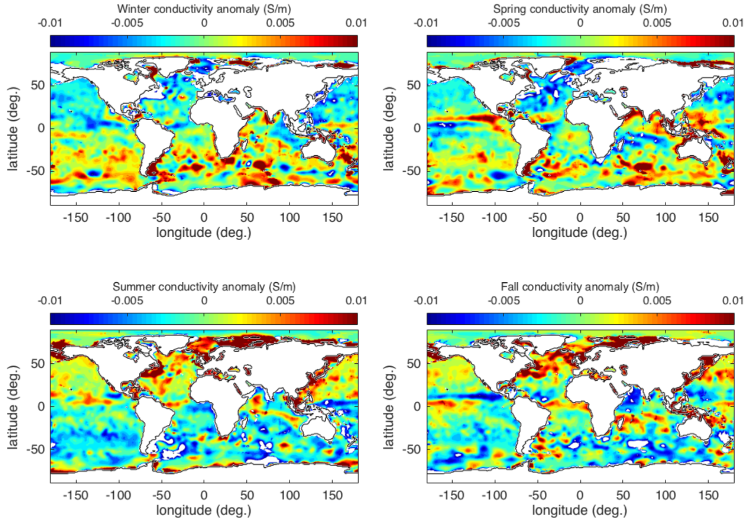

Fig S1: Depth-averaged conductivity anomaly (relative to annual mean) for each of the four seasons.The full range in the data are, respectively, -0.660—0.556 (S/m), -0.471—0.317 (S/m), -0.511—0.721 (S/m), -0.502—0.594 (S/m).
