# Supplementary material for: Electrical conductivity of the global ocean
Source: Earth Planets Space. 2017 Nov 14;69(1):156. doi: 10.1186/s40623-017-0739-7 (PMC6959386; doi:10.1186/s40623-017-0739-7)
Supplement: Supplementary file 2 — Additional file 2. Supplement document comparing results here with results from a previous study. [file 40623_2017_739_MOESM2_ESM.docx]

*Earth, Planets and Space*

Supporting Information for

Electrical Conductivity of the Global Ocean

Robert H. Tyler^1,2^, Tim P. Boyer^3^, Takuto Minami^4^, James R. Reagan^3,5^, Melissa M. Zweng^3^

^1^NASA Goddard Space Flight Center, Greenbelt, Maryland, USA

^2^Astronomy Department, University of Maryland, College Park, MD, USA

^3^National Centers for Environmental Information (NCEI), National Oceanic and Atmospheric Administration (NOAA), Silver Spring, MD, USA

^4^Earthquake Research Institute, The University of Tokyo, 1-1-1 Yayoi, Bunkyo-ku, Tokyo, 113-0032, JAPAN

^5^Cooperative Institute for Climate and Satellites (CICS), Earth System Science Interdisciplinary Center (ESSIC) University of Maryland, College Park, MD, USA

**Conductivity Climatology – Derivation through two different methods:**

Conductivity in the ocean is a function of three variables: temperature (T), salinity (S), and pressure (P). For this study we introduce a conductivity climatology derived from conductivity calculated from each oceanographic cast in the World Ocean Database (Boyer *et al.* 2013) that contains concurrently measured temperature and salinity. This yields profiles of calculated conductivity that are then mapped onto a global grid through the objective analysis scheme employed in the creation of other World Ocean Atlas 2013 (WOA13) fields (Locarnini *et al.* 2013, Zweng *et al.* 2013). This “in situ method” (hereafter ISM), is different from the “climatology derived method” (hereafter CDM), that Manoj *et al.* (2006) used in which they took previously gridded climatologies of temperature and salinity and calculated a conductivity climatology based off of this.

The climatological T, S data in the WOA13 (Locarnini *et al.* 2013, Zweng *et al.* 2013) and previous releases of WOA, such as WOA01, which was used in the Manoj *et al.* (2006) study, represent spatial/temporal distributions of observed T, S but the distributions of observed T, S data are somewhat different because some observations reported only one of either T or S. While climatologies of either T or S can be obtained through objective analyses of the respective T, S data available, problems can arise when one attempts to combine these data derived from different temporal/spatial distributions of original data. This is especially a concern in the calculation of conductivity which involves a nonlinear dependence on T, S. The CDM may indeed give conductivity values that are neither representative nor realistic. The ISM requires coincident, co-located T, S observations and therefore avoids these unrealistic results.

The ISM is the preferred method when compared to the CDM because it utilizes calculated conductivity profile data from concurrent measurements of temperature and salinity, whereas the CDM does not. However, the advantage to using the CDM is that it can be calculated much faster than the ISM. Thus, a comparison analysis between results from these two approaches is undertaken to examine how large the differences are and what impacts they can potentially have. The comparison will use an ISM conductivity climatology from 1981-2010 and a CDM conductivity climatology calculated from temperature and salinity climatologies for the same time period. The years 1981-2010 were chosen as this is a common 30 year ‘climate normal’ period used in many studies (WMO,2011) and because most, if not all, salinity values will have used the Practical Salinity Scale 1977 (PSS77, generally adopted by 1981). Some previous applications using the Manoj *et al.* (2006) data (i.e., CDM) involved solutions of the electromagnetic induction equation for frequencies where the electromagnetic wavelengths in seawater are much larger than the ocean depth. In such cases, the solutions are sensitive only to the integrals of conductivity and conductivity/flow velocity products. Thus, the first portion of this comparison analysis focuses on the depth-integrated conductivity (i.e., conductance) differences.

Figure 1a-d represents the 1981-2010 annual conductance climatology comparison between the two derivation methods. The percent error, defined as the difference of the CDM value and the ISM value at each grid point divided by the full ISM value at that grid point, is less than one percent (Figure 1d) over most of the global ocean. The percent error is greater than one percent in coastal locations and in the Arctic. Both of these locations exhibit very low conductance values, either due to shallow depths and/or cold/fresh water, and thus a higher percent error should be expected. Most of these regions also suffer from poor data coverage. The CDM uses all temperature and salinity data, regardless of whether or not they were concurrently observed. This can have repercussions as the mean temperature and salinity may not necessarily reflect the same ocean.

Figure 2a-b represents the 1981-2010 seasonal cycle amplitude of the 0-1500m conductance as derived from the first harmonic of the Fourier decomposition for both conductivity derivation methods. The monthly conductance fields, for which the seasonal cycle was derived, have a depth limit of 1500m which is why Figure 2 shows the seasonal cycle amplitude of only the upper 1500m. The seasonal cycle below 1500m is less than 20S over ~95% of the global ocean, therefore it is excluded from this analysis. The 0-1500m seasonal cycle amplitudes are also quite small. They reach nearly 100S in some areas, but this still only represents 1-2% of the full conductance value (see Figure 1a,b). Figure 2c and 2d show the difference and the percent error of the seasonal cycle amplitudes from both methods, respectively. Large percent errors (Fig. 2d) persist mainly in regions where there is very little seasonal cycle (e.g., Arctic) which is expected. However, in the vicinity of the Antarctic Circumpolar Current (and associated fronts) there is a clear circumpolar region of high (> 50%) percent errors. Dong *et al.* (2006) showed that while the Antarctic Polar Front (PF) does not have much seasonally spatial movement, the temperature along the PF does have a clearly defined seasonal cycle. Thus, it is expected that concurrent measurements of T and S, a requirement of the ISM, would capture the volatility of the front, and thus its seasonal cycle much better than temperature and salinity measurements that could be taken separately at different times as in the CDM.

Thus far we have only compared annual conductance and the seasonal cycles of conductance utilizing the two derivation methods. However, when conductance is used in the induction equation, the accuracy of the gradients of conductance and the gradients of the inverse of conductance are equally important as the full values of conductance. Therefore, we compare the CDM and ISM zonal gradients (Figure 3) and meridional gradients (Figure 4) of conductance. Additionally, the zonal and meridional gradients of the inverse of conductance are also compared in Figures 5 and 6, respectively. It is clear that in Figures 3 through 6, the gradients are driven mostly by the ocean’s bottom topography. Large percent errors in Figures 3d through 6d are scattered throughout the global ocean and are confined mainly to regions of very weak conductance gradients, thus even a very small difference between the two methods would yield large percent errors. Additionally, the Arctic experiences very different conductance zonal gradients as shown in Figures 3 and 5 between the two conductivity derivation methods. Unlike the rest of the global ocean, where large percent errors were confined to weak zonal gradients, the Arctic experiences large differences even in regions of large zonal gradients (Figs. 3d and 5d).

Figures 1 through 6 show that over most regions the CDM and ISM results are very similar when comparing conductance and the gradients of conductance. However, as noted previously, conductance and the gradients of conductance are largely influenced by ocean depth while the point of the analysis specifically concerns the comparison of conductivity distributions. Thus, a comparison analysis of depth-averaged conductivity was also undertaken. Due to conductance being equal to the depth-averaged conductivity multiplied by the bottom depth, Figure 1d also represents the percent error in the depth-averaged conductivity comparison because the bottom depths are identical in both the ISM and CDM. Thus, by examining Figure 1d, we see that depth-averaged conductivity is very similar in both methods with percent errors greater than 1% in the Arctic, coastal regions, and some inland seas.

While the full values of conductance and depth-averaged conductivity are directly related through ocean topography; the gradients of conductance are more influenced by ocean topography than are the gradients of depth-averaged conductivity. Thus, it is expected that the depth-averaged conductivity gradients would yield a more accurate assessment of how well the ISM and CDM compare. Therefore, Figures 7 and 8 illustrate the comparison of the two derivation methods for the zonal and meridional gradients of depth-averaged conductivity, respectively. While many of the gradients are still topographically influenced in Figures 7a,b and 8a,b, the percent errors of depth-averaged conductivity gradients (Figs. 7d and 8d) as compared to the percent errors of conductance gradients (Figs. 3d and 4d) are much larger and more widespread. The regions of high percent difference from Figs. 3d and 4d still exist in Figs. 7d and 8d; however, they are expanded with many more locations experiencing large (>20% error) gradient differences in depth-averaged conductivity between the two derivation methods. This is at least partially due to the comparison of very small zonal (Figs. 7a,b) and meridional (Figs 8a,b) depth-averaged conductivity gradients. However, even taking this into account, there is still a vast spatial increase of large differences (>20% error) in Figs. 7d and 8d. The ISM/CDM comparison analysis of the inverse of the depth-averaged conductivity of zonal (Fig. 9d) and meridional (Fig. 10d) gradients show similar increases in percent differences when compared to the zonal gradient (Fig. 5d) and meridional gradient (Fig. 6d) of the inverse of conductance. Thus, while the analysis of conductance gradients yielded mainly weak and sparse differences between the ISM and CDM, the analysis of depth-averaged conductivity gradients painted a different picture with many more regions experiencing large differences between the two derivation methods.

There are some regions with persistent conductance and depth-averaged conductivity differences, such as the Arctic Ocean, shallow coastal regions, and marginal/inland seas. Figure 11 highlights an example of why the ISM is preferable to the CDM, where temperature and salinity data distribution differences matter.

Figure 11 shows the May climatological mean conductivity profiles in a box centered at 6.5S, 133.5E, in an area between Australia and New Guinea. The red profile is from ISM conductivity. The green profile is CDM conductivity. Both are for the time period 2005-2012. The green profile shows significantly higher values than the red over most depths. The reason for the larger values of conductivity calculated from the CDM is that the climatological temperature was calculated based on XBT (eXpendable BathyThermograph) data from two cruises—one in 2005, the other in 2009. There are no salinity data for the time period 2005-2012 in the area. Thus, the climatological temperature for the area for 2005-2012 is based on data from the time period, whereas the climatological salinity defaults to a combination of the longer time period salinity (the first-guess field) and salinity data outside the marginal sea, for which there are salinity data in the area during 2005-2012. This mismatch between the climatological temperature and salinity creates a conductivity field for the area which is representative of the temperature from 2005-2012, but not the salinity. The conductivity cannot be calculated directly from observations for the 2005-2012 time period due to the lack of salinity data, so the area conductivity defaults to the 1981-2010 conductivity modified by conductivity outside the marginal sea, which is consistent since there are both temperature and salinity data measured concurrently. The 1981-2010 conductivity climatology, which does have good sampling (at least the best available) in these areas, is used as a first-guess field for the analyzed 2005-2012 field, so these areas are well represented in the latter. This is not a perfect solution, since the analyzed conductivity will be strongly influenced by the 1981-2010 conductivity for the area and the 2005-2012 conductivity outside the marginal sea. Still, it is a better solution than a conductivity profile which is not physically realistic. For the open ocean during 2005-2012, Argo is the main observing system, collecting both temperature and salinity data. But there are many areas (marginal seas, boundary currents, even some open ocean areas) for which temperature-only measurements are more prevalent. Further, different quality control procedures are performed on temperature and salinity for Argo floats, leading to profiles of temperature in an area which passed quality control, but salinity profiles which did not pass, again leading to an inconsistency in the temperature and salinity climatologies. While it is optimal to include as many data as possible in the separate temperature and salinity climatologies, the separate climatologies should not be used to calculate a conductivity climatology directly. It should be noted that the 2005-2012 WOA13 temperature and salinity climatologies (used in calculating the 2005-2012 CDM climatology) used a first-guess field of 1955-2012, while the 2005-2012 ISM conductivity climatology used 1981-2010 to ensure the use of only Practical Salinity Scale data.  The difference in first-guess fields results in a CDM conductivity error (not shown) of < 0.05S/m (usually much less) which is much smaller than the differences seen between ISM and CDM in Figure 11.

Figure 11 highlights the main issue with using the CDM to derive conductivity. Historically, the number of temperature profiles taken has greatly outnumbered that of salinity in the World Ocean Database (see Figure 12). This gap started closing with the advent of the Argo profiling program in the late 1990’s. However, before this time, temperature dominated salinity in global spatial distribution of observations. Thus, there were many regions of the ocean where temperature was sampled during a particular time period, but salinity was not. Thus, when computing the conductivity climatology over a certain period, for example, 1981-1990, using the CDM, many regions would have temperature data over that decade, but no salinity. Therefore, the correct decadal temperature would be used in calculating conductivity; however, the salinity would not be 1981-1990 salinity, but rather the long-term mean (i.e., first-guess field) which would yield unrepresentative conductivity results for that decade. Additionally, it should be noted that Manoj *et al.* (2006) used the World Ocean Atlas 2001 in their study, which lacked much of the Argo profiling data that is now in the World Ocean Database (see Figure 12). Thus, even the long-term mean of conductivity calculated with WOA01 lacked sufficient global coverage of salinity profiles, particularly in the Southern Ocean, and therefore is subject to great uncertainty in certain regions.

Based on this comprehensive comparison analysis, the authors recommend that the ISM climatology of conductivity revealed in this study be used for conductivity-related research as it does not suffer from the inherited weaknesses of the CDM conductivity climatology derived from climatological temperature and salinity.

**References:**

1. Boyer, T.P., J. I. Antonov, O. K. Baranova, C. Coleman, H. E. Garcia, A. Grodsky, D. R. Johnson, R. A. Locarnini, A. V. Mishonov, T.D. O'Brien, C.R. Paver, J.R. Reagan, D. Seidov, I. V. Smolyar, and M. M. Zweng, 2013: World Ocean Database 2013, ***NOAA Atlas NESDIS 72,*** S. Levitus, Ed., A. Mishonov, Technical Ed.; Silver Spring, MD, 209 pp.
2. Dong, S., J. Sprintall, and S. T. Gille, 2006: Location of the Antarctic Polar Front from AMSR-E satellite sea surface temperature measurements, *J. Phys. Oceanogr.*, **36**, 2075–2089.
3. Locarnini, R. A., A. V. Mishonov, J. I. Antonov, T. P. Boyer, H. E. Garcia, O. K. Baranova, M. M. Zweng, C. R. Paver, J. R. Reagan, D. R. Johnson, M. Hamilton, and D. Seidov, 2013: ***World Ocean Atlas 2013, Volume 1: Temperature***. S. Levitus, Ed., A. Mishonov Technical Ed.; ***NOAA Atlas NESDIS 73***, Silver Spring, MD, 40 pp.
4. Manoj, C., A. Kuvshinov, S. Maus, and H. Lühr, 2006: Ocean circulation generated magnetic signals, *Earth Planets Space*, **58**(4), 429–437.
5. WMO, 2011: World Metoroilogical Organization: Guide to Climatological Practices, 2011 Edition, WMO, Geneva, Switzerland.
6. Zweng, M.M, J.R. Reagan, J.I. Antonov, R.A. Locarnini, A.V. Mishonov, T.P. Boyer, H.E. Garcia, O.K. Baranova, D.R. Johnson, D.Seidov, and M.M. Biddle, 2013: ***World Ocean Atlas 2013, Volume 2: Salinity***. S. Levitus, Ed., A. Mishonov Technical Ed.; ***NOAA Atlas NESDIS 74***, Silver Spring, MD, 39 pp.

**Figures:**

Figure 1: 1981-2010 annual conductance derived from a) climatology derived method (CDM), b) in situ method (ISM), c) CDM-ISM, and d) the percent error [(CDM-ISM)/ISM]*100.


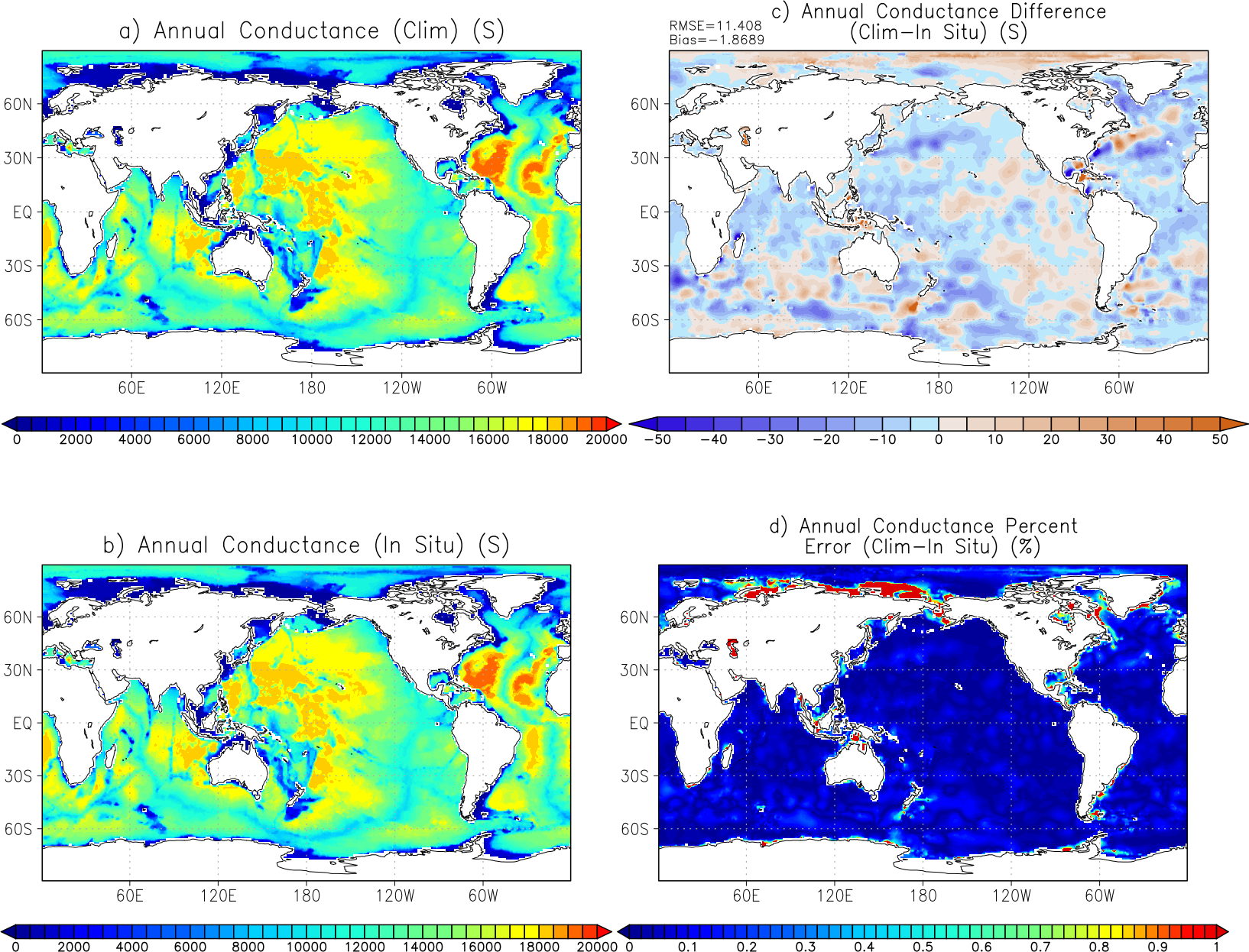


Figure 2: 1981-2010 amplitude of the seasonal cycle (first harmonic) of conductance derived from a) climatology derived method (CDM), b) in situ method (ISM), c) CDM-ISM, and d) the percent error [(CDM-ISM)/ISM]*100.


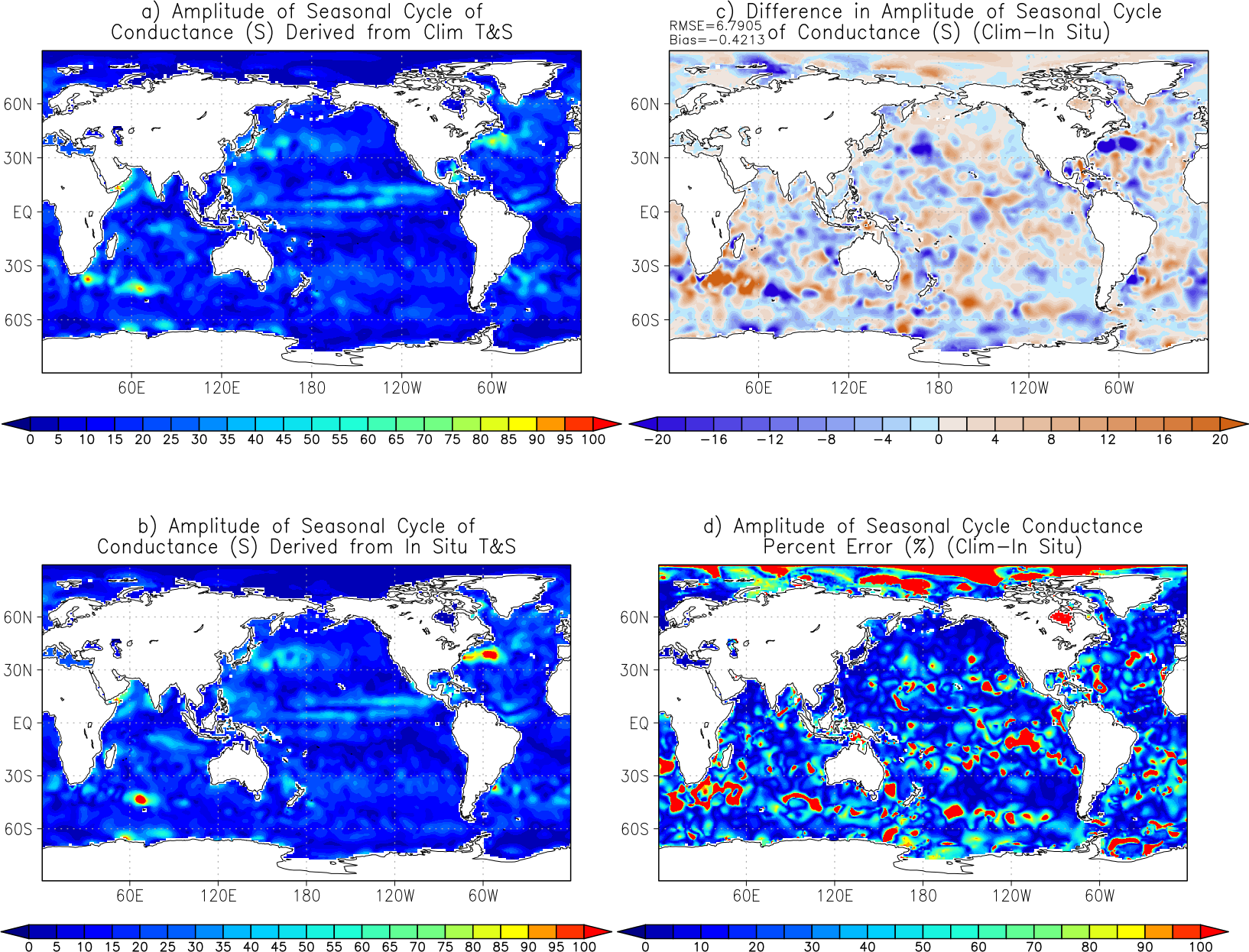


Figure 3: 1981-2010 zonal gradient of the annual conductance derived from a) climatology derived method (CDM), b) in situ method (ISM), c) CDM-ISM, and d) the percent error [(CDM-ISM)/ISM]*100.


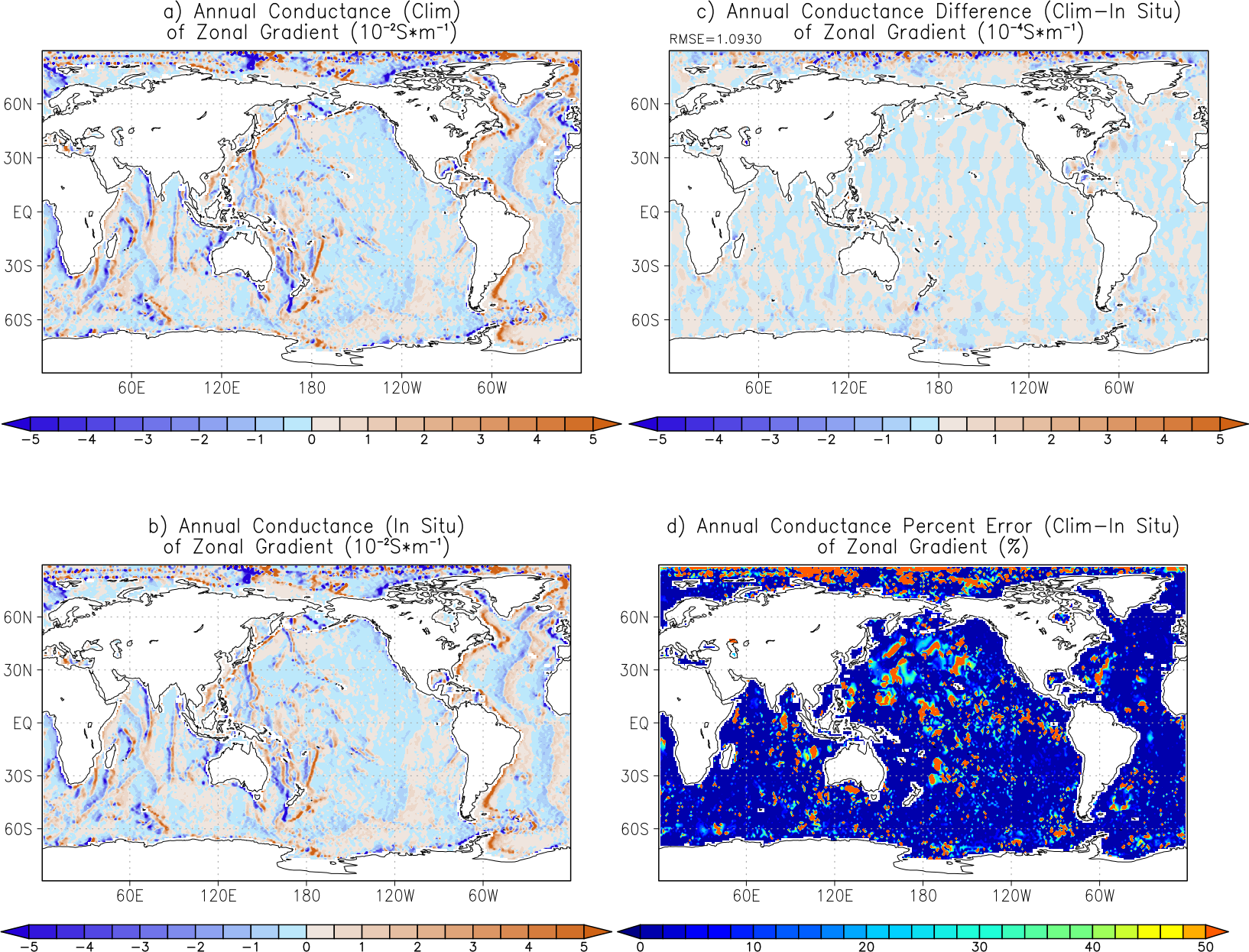


Figure 4: 1981-2010 meridional gradient of the annual conductance derived from a) climatology derived method (CDM), b) in situ method (ISM), c) CDM-ISM, and d) the percent error [(CDM-ISM)/ISM]*100.


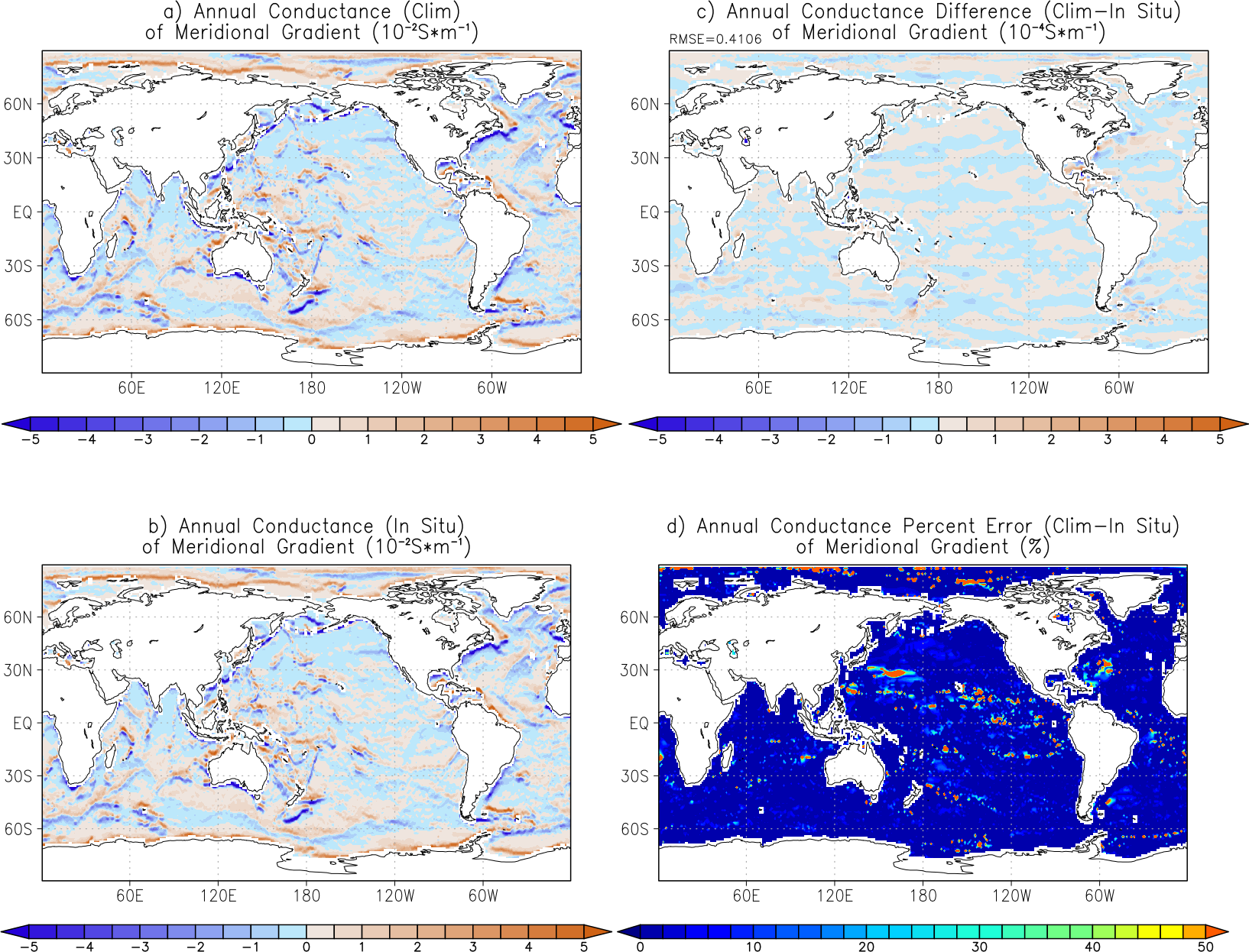


Figure 5: 1981-2010 zonal gradient of the inverse of the annual conductance derived from a) climatology derived method (CDM), b) in situ method (ISM), c) CDM-ISM, and d) the percent error [(CDM-ISM)/ISM]*100.


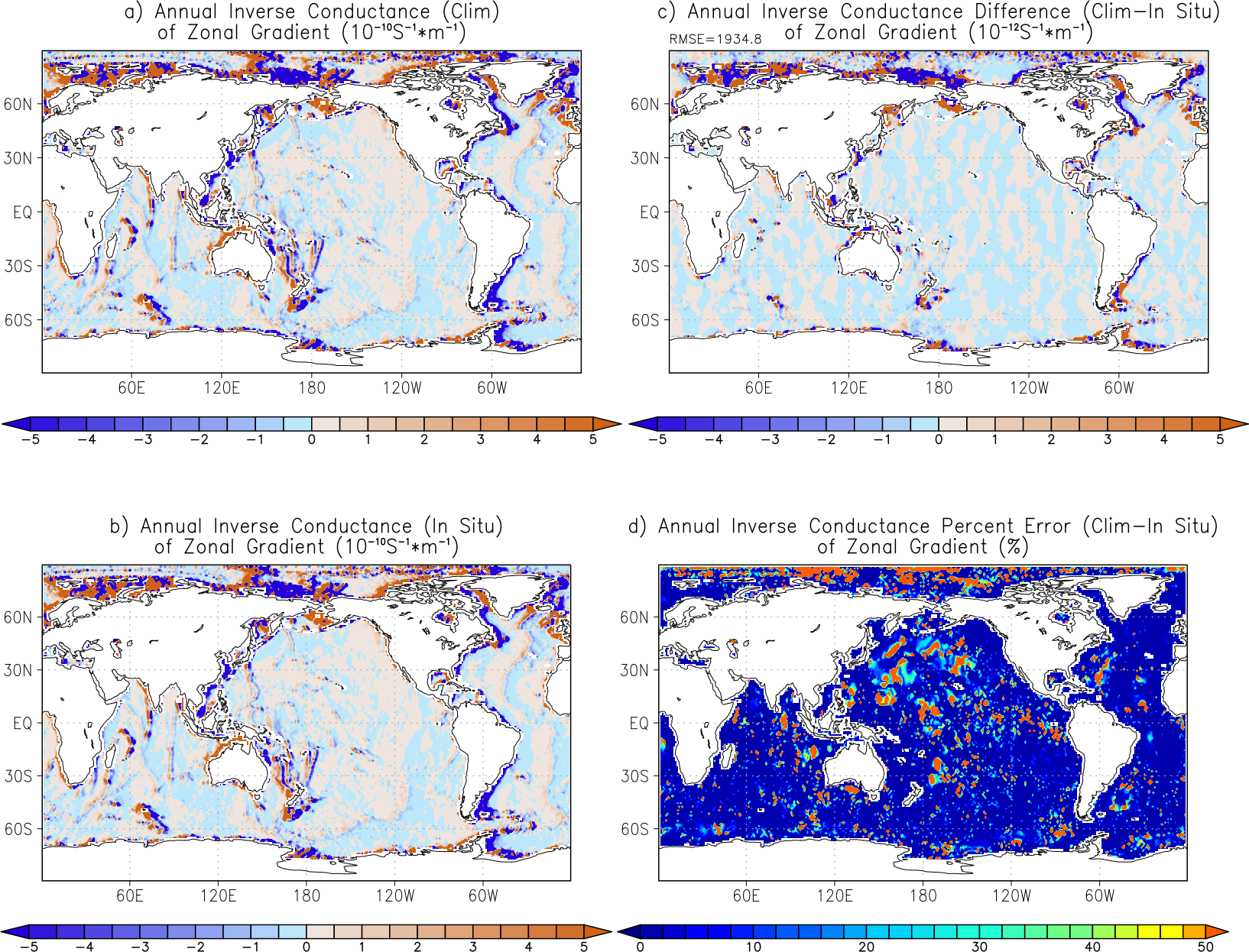


Figure 6: 1981-2010 meridional gradient of the inverse of the annual conductance derived from a) climatology derived method (CDM), b) in situ method (ISM), c) CDM-ISM, and d) the percent error [(CDM-ISM)/ISM]*100.


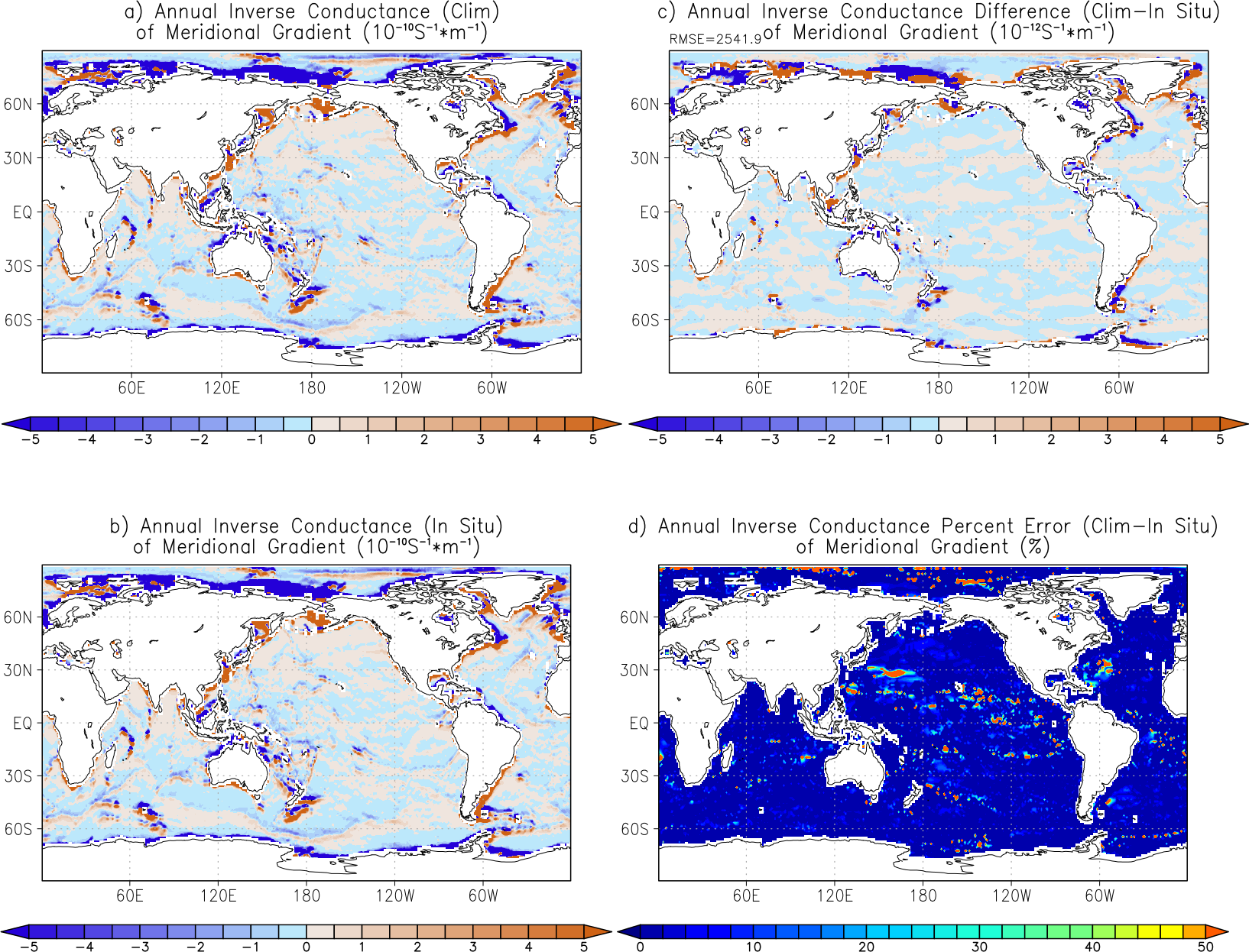


Figure 7: 1981-2010 zonal gradient of the annual depth-averaged conductivity derived from a) climatology derived method (CDM), b) in situ method (ISM), c) CDM-ISM, and d) the percent error [(CDM-ISM)/ISM]*100.


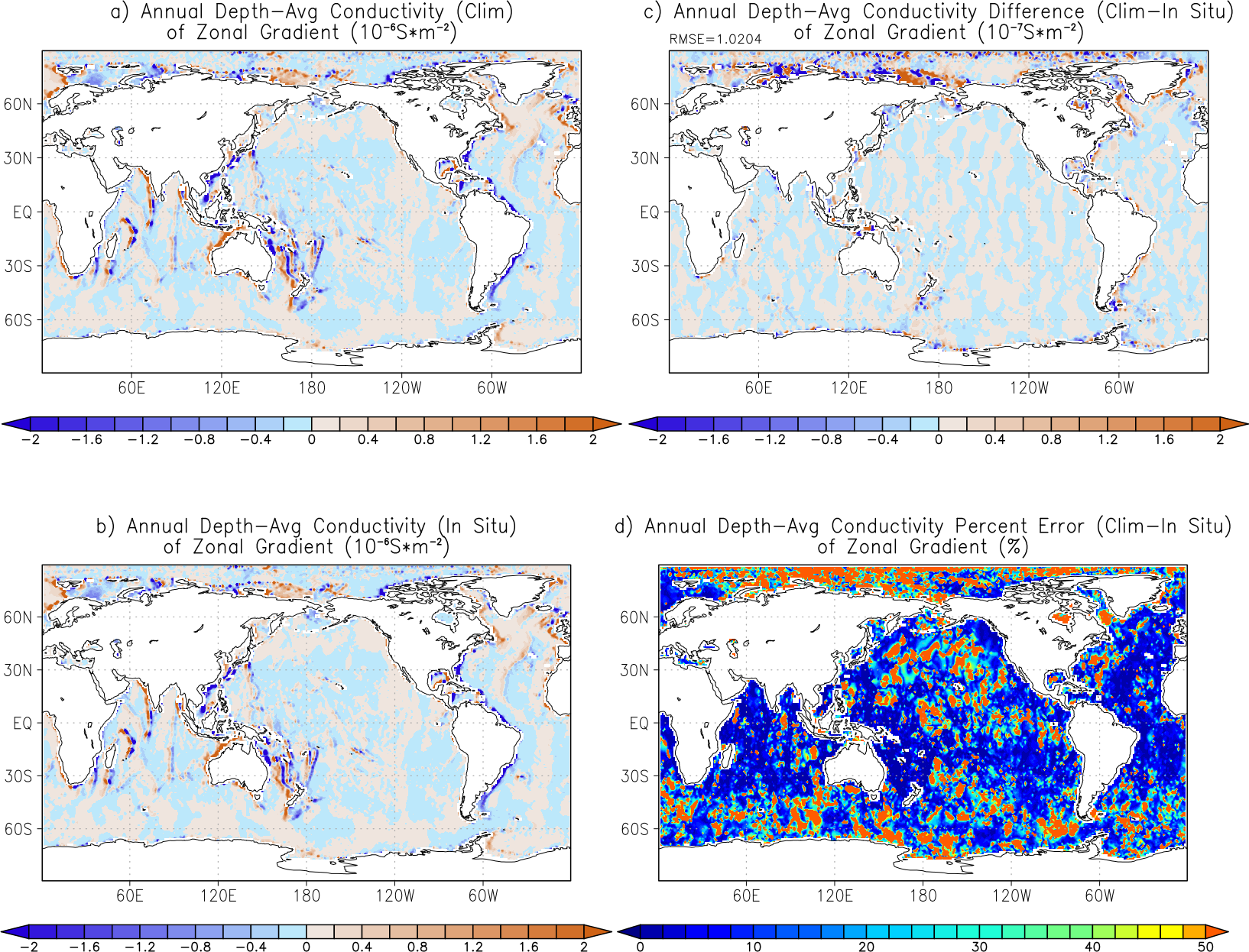


Figure 8: 1981-2010 meridional gradient of the annual depth-averaged conductivity derived from a) climatology derived method (CDM), b) in situ method (ISM), c) CDM-ISM, and d) the percent error [(CDM-ISM)/ISM]*100.


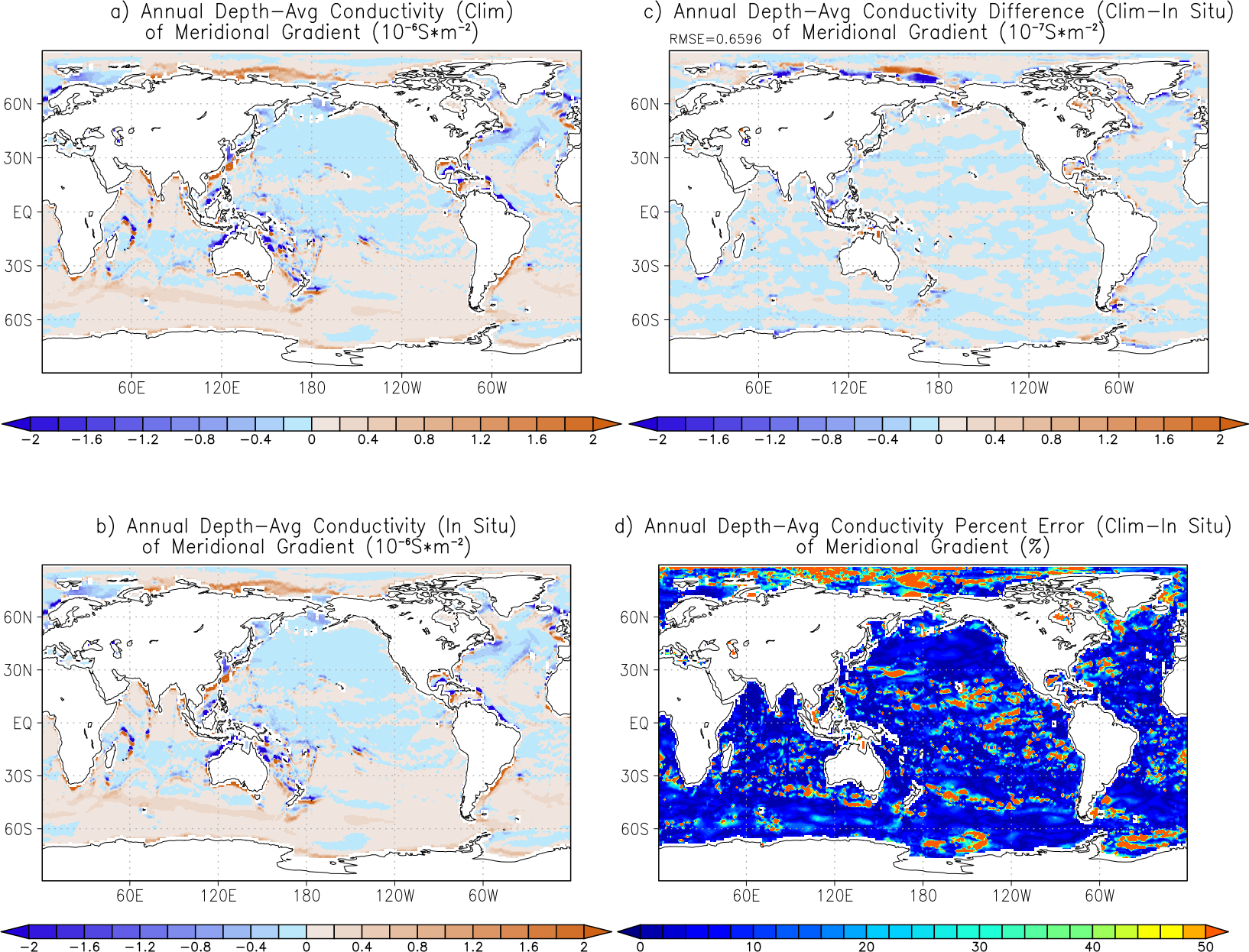


Figure 9: 1981-2010 zonal gradient of the annual inverse depth-averaged conductivity derived from a) climatology derived method (CDM), b) in situ method (ISM), c) CDM-ISM, and d) the percent error [(CDM-ISM)/ISM]*100.


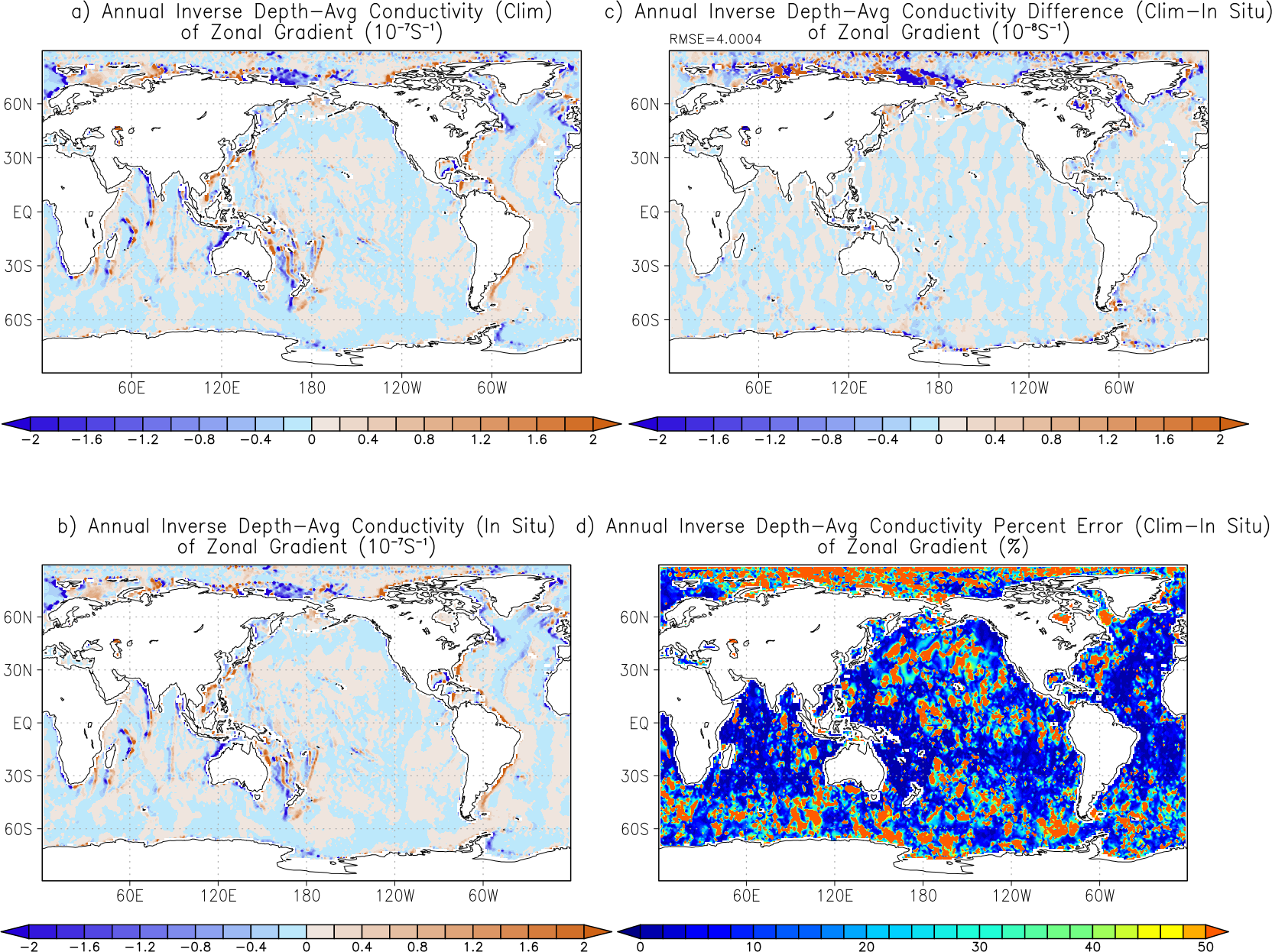


Figure 10: 1981-2010 meridional gradient of the annual inverse depth-averaged conductivity derived from a) climatology derived method (CDM), b) in situ method (ISM), c) CDM-ISM, and d) the percent error [(CDM-ISM)/ISM]*100.


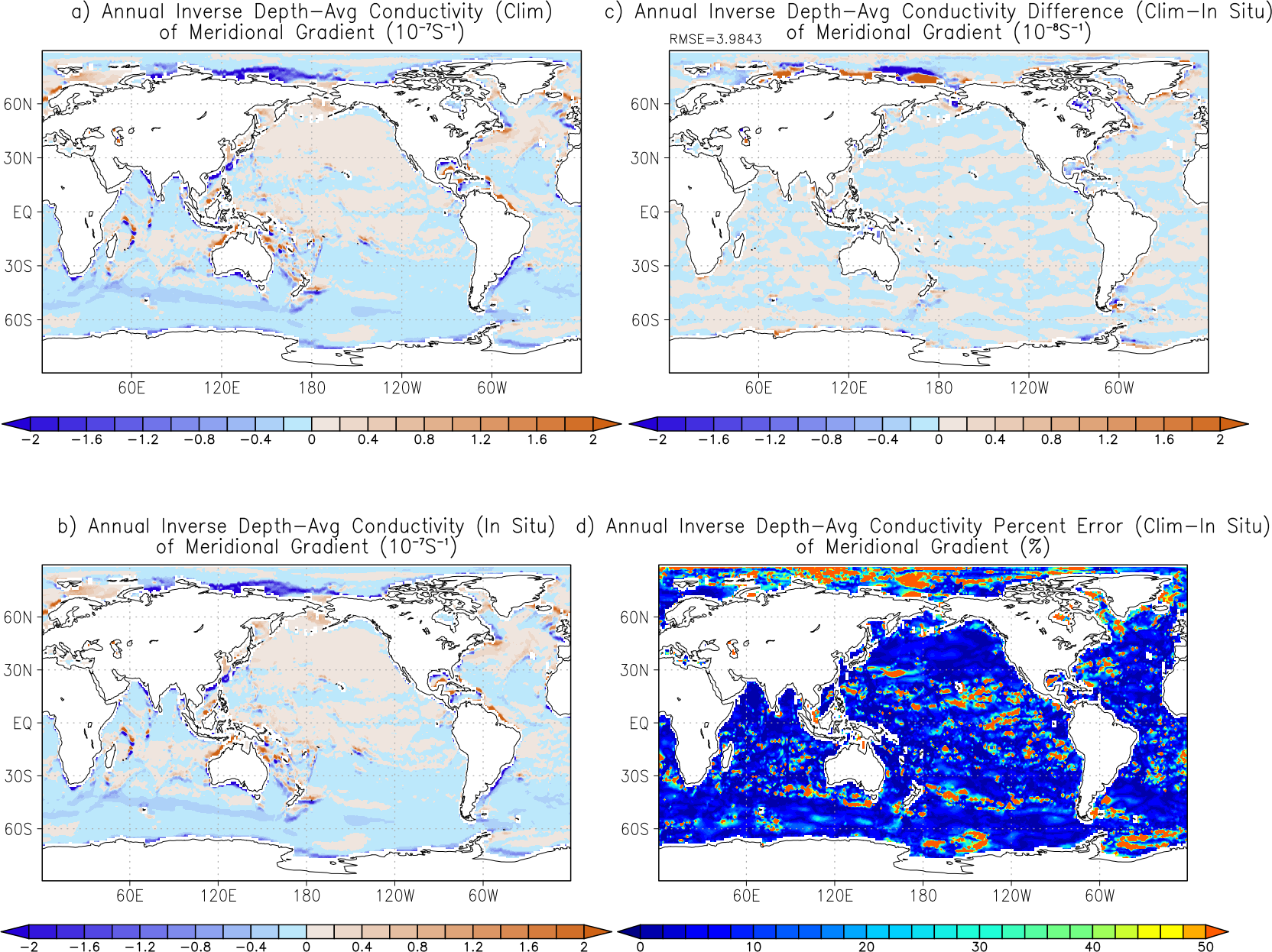


Figure 11: May 2005-2012 conductivity in a one-degree by one-degree box centered at 133.5E, 6.5S from the in situ method (ISM, red) and from the climatology derived method (CDM, green). The large and systematic errors seen in some regions using the CDM are compounded when depth-integrating to obtain the geophysically important conductance.


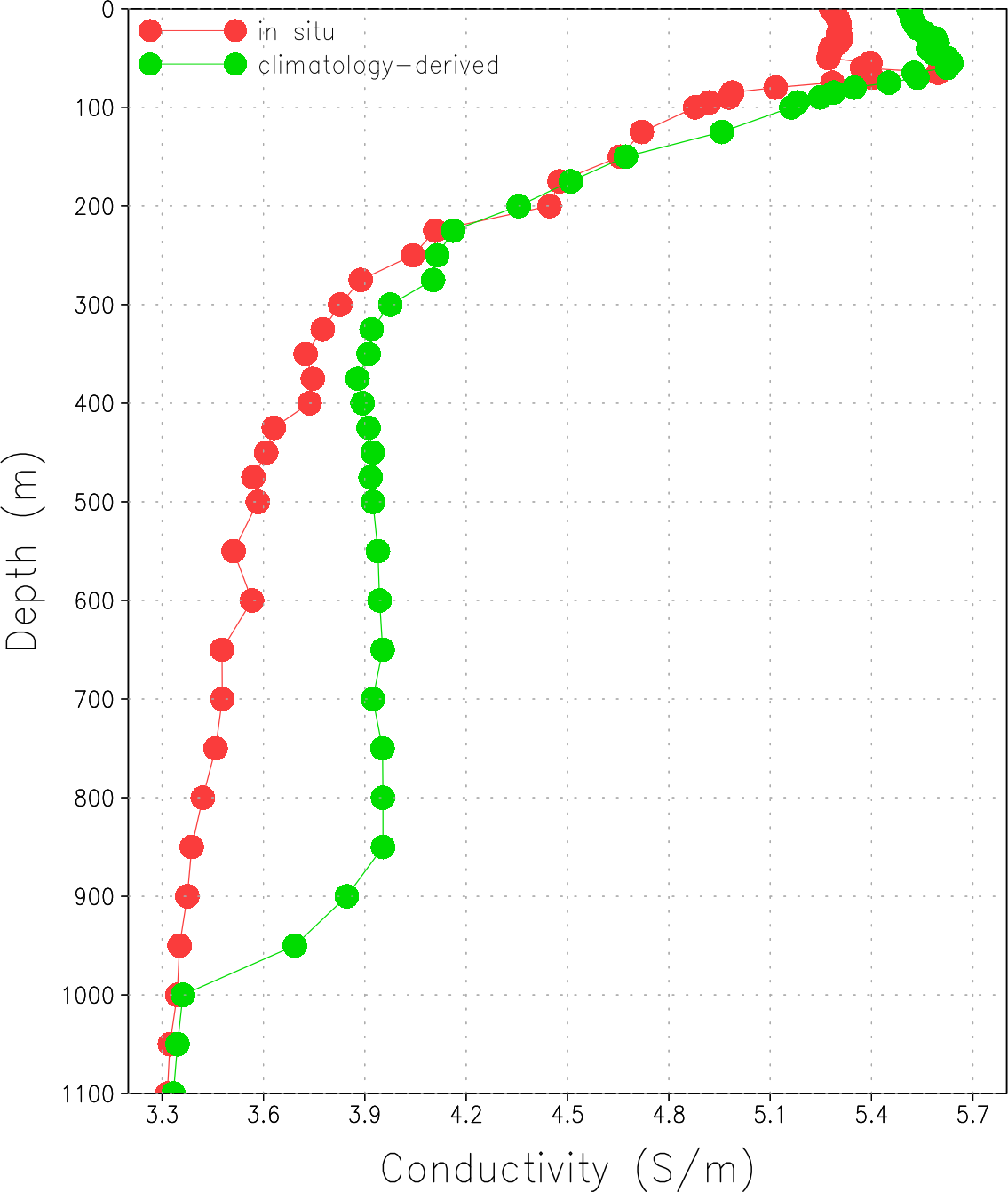


Figure 12: Number of temperature and salinity measurements for each pentad (5-year) from 1981 to 2010 in the World Ocean Database. This includes temperature and salinity data from bottles, CTDs, XBTs, MBTs, profilers, undulating oceanographic recorders, drifting buoys, and gliders. It does not include moored buoys, surface-only (e.g., TSG), or autonomous pinniped bathythermograph (APB) data.
